# Dispersion characteristics of radioactive materials estimated by wind patterns

**DOI:** 10.1038/s41598-018-27955-4

**Published:** 2018-07-02

**Authors:** Takao Yoshikane, Kei Yoshimura

**Affiliations:** Institute of Industrial Science, The University of Tokyo, 5-1-5, Kashiwanoha, Kashiwa-shi, Chiba 277-8574 Japan

## Abstract

The radioactive materials are generally concentrated downwind of their origins when the prevailing winds blow continuously in one direction. If this principle determined the pattern of dispersion in all cases, dispersion directions could be estimated by wind patterns. However, this hypothesis has not been sufficiently verified because of the complexity of dispersion processes and weather systems. Here, we show that dispersion directions, which are divided into four ranges, can be estimated by wind patterns using a machine learning approach. The five-year average hit rates of the directions of dispersion estimated using near-surface winds exceed 0.85 in all months. The dispersion directions can be estimated up to 33 hours in advance using forecast winds. In particular, high hit rates exceeding 0.95 are achieved in January and March, when large-scale weather systems dominate. These results indicate that the dispersion directions are determined by the wind patterns that correspond to large-scale weather systems and diurnal circulation patterns in most cases. Our findings also provide more reliable information on dispersion patterns with reduced uncertainties, given that reasonable skill is achieved at a sufficient lead time for evacuation.

## Introduction

High contamination densities of ^137^Cs exceeding 1480 kBqm^−2^ were observed from 150 to 250 km northeast of the Chernobyl nuclear power plant after the accident in April 1986^1–6^. The details of the dispersion process are still largely unknown. However, these observations indicate that large amounts of radioactive materials travelled long distances (exceeding one hundred km) from the emission source in this accident. The dispersion of radioactive materials also depends on emission height because wind speeds and directions change vertically. These materials could reach heights up to 2000 m or even greater over short periods, due to the high temperatures of the melting core in the first two days of the accident^2^. Some of the radioactive materials were transported all over the world by westerly winds in the middle to upper troposphere after they had been lifted by upward winds produced by deep convection^7^. However, the remainder was deposited in the regions neighbouring Chernobyl after these materials were transported by lower tropospheric winds^1–3,8^. Lower winds also had strong effects on the dispersion of radioactive materials released during the Fukushima Daiichi nuclear power plant accident in March 2011^9,10^. Local winds (e.g., land-sea breezes and mountain-valley winds) also influenced the dispersion of this material.

Numerical models of the atmosphere have been developed to represent dispersion processes and weather systems with various scales ranging from local circulation patterns to monsoons. In the Fukushima Daiichi nuclear power plant (FDNPP) accident in March 2011, predictions of the dispersion of radioactive materials made using atmospheric models were not used to inform evacuations^11–13^. It has been reported that the models did not perform satisfactorily because the emission source information was lost due to power failures^13^. The Japanese government did not release the predictions to the public until 23 March because of a lack of reliability^11^. This decision led to public concern and caused confusion^13^. Afterwards, the investigation committee reported that the government could have used the simulation results efficiently, even though the emission source information had been lost^13^. They advised that the result of simulation made assuming unit emissions (a fixed release rate) should be used to inform evacuations. The characteristics of dispersion patterns, such as direction and range, which are influenced by the winds that accompany weather systems, can be estimated in such experiments, although the amounts of nuclear materials deposited cannot be estimated because they depend strongly on the emission conditions. Experiments assuming unit emissions may be useful for risk management, including evacuations, in that they determine the dispersion patterns that correspond to the prevailing weather conditions.

Despite the committee’s advice, the Japanese government has decided not to use model predictions to determine whether people should evacuate in the future because such model predictions have limited accuracy^14,15^. However, this decision may increase the risk of radiation exposure in cases in which limited information is available. For example, the window of opportunity for taking potassium iodide (KI), which must be taken 48 hours or less before ^131^I exposure^16^, might be missed. To reduce the risk of exposure, the government should give warnings regarding the consumption of water and the distribution of farm products as early as possible when radioactive deposition occurs^17,18^. However, taking appropriate action is challenging if no predictions are available.

Simply showing the result of high-resolution simulation might also be misunderstood because such predictions give the impression that the model can represent the dispersion distribution very precisely. Explaining the reliability of predictions is challenging. This fact may partly explain why policy makers have chosen not to use model predictions to inform evacuation decisions.

Admittedly, we cannot avoid the prediction errors that result from imperfect model formulations or the sensitive dependence of such models to initial conditions^9,19,20^. In fact, several high-performance models failed to represent the radioactive plumes passing through a given area, and the arrivals of the simulated radioactive plumes were advanced or delayed by several hours in some areas from 100 to 300 km south-southwest of the FDNPP^19^. In general, model uncertainties are estimated by performing massive ensemble experiments, which produce probabilistic forecasts. However, it is usually difficult to explain the uncertainty to laypeople^20^. In an emergency in which serious damage may occur, reliable information is required, rather than a probabilistic forecast that includes uncertainties. Therefore, a significant reduction in uncertainties is necessary to obtain more reliable predictions from simulations performed using numerical models. The identification of regular patterns or generalizations of complex phenomena could be an effective approach in both reducing uncertainties and clearly explaining the reliability of predictions to laypeople.

In general, radioactive materials become concentrated on the downwind sides of their sources when the prevailing winds blow in one direction continuously. For example, the risk of exposure is expected to be higher on the downwind side of a source of radioactive material compared to the upwind side when large-scale weather systems dominate. If this simple principle is the most important factor that determines the pattern of dispersion under all weather conditions, dispersion directions can be determined using wind patterns. The risk of exposure could then be estimated using the wind conditions. However, whether this hypothesis is true remains unclear because the dispersion processes and weather systems are quite complex. In this study, a new machine learning-based prediction method is applied to clarify the dispersion patterns corresponding to the wind patterns resulting from complex weather systems during specific months (January, March, April, July, and October), which are representative of each season.

## Results

**Figure 1:**
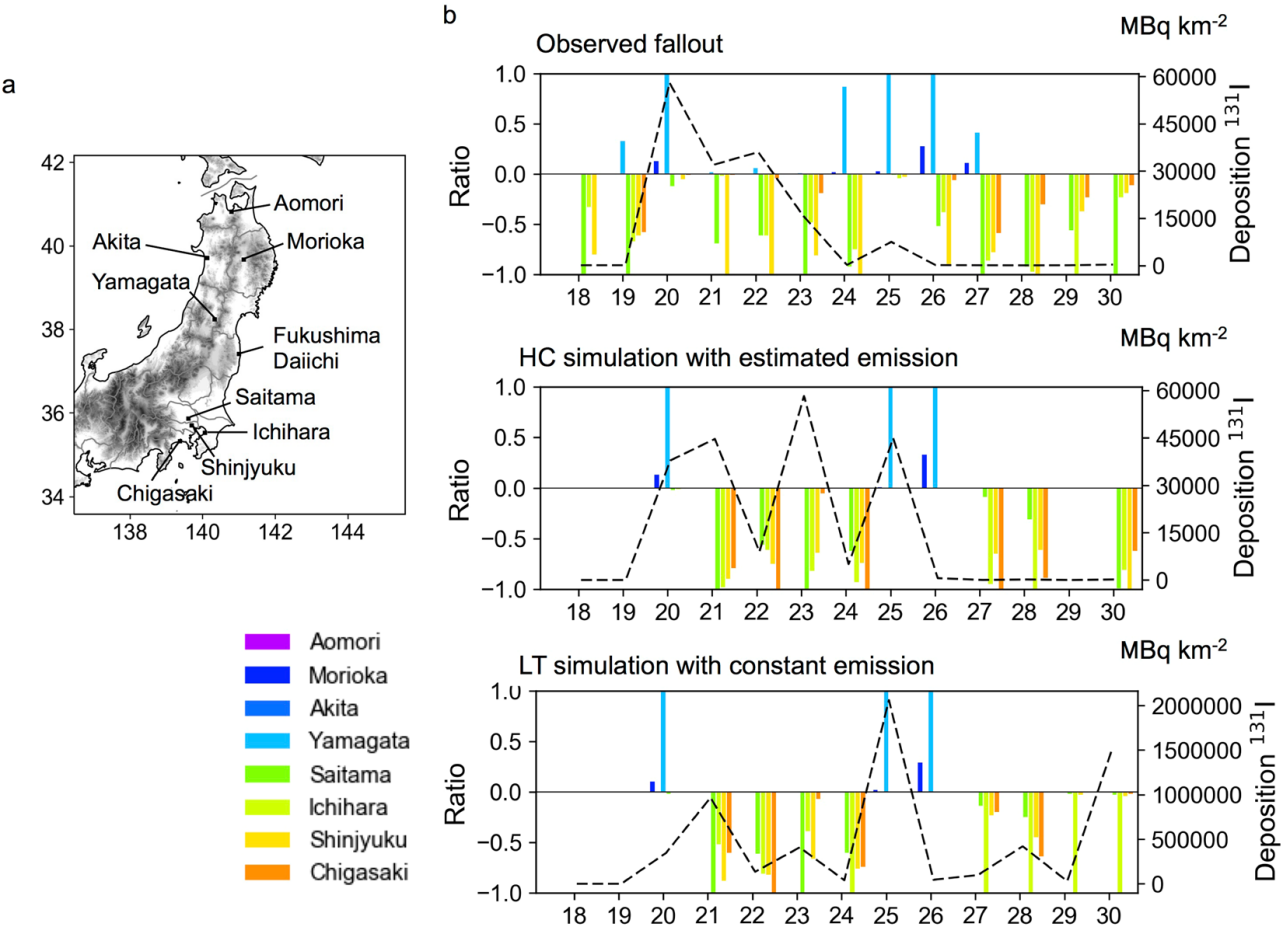
Meridional directions of disposition of ^131^I (**a**). The sites where the daily cumulative deposition of radioactive materials was observed (Nuclear Regulation Authority, Japan). (**b**) Temporal variations of the observed and simulated deposition amounts in March 2011. The bars indicate the ratio of the deposition at each point to the maximum among the eight points and the dashed lines are the maximum daily deposition among the eight points. Four of the points are located to the north of the FDNPP (positive values), whereas the others are located to the south of the FDNPP (negative values). The map and graphs were created using Python 3.6.

The daily variations in the deposition of a specific radioactive material, ^131^I, are shown in Fig. 1. A meridional dispersion pattern can be clearly identified in the daily deposition patterns, except on the days when the direction of dispersion changes drastically. Almost no deposition is noted in the northern area when large amounts of ^131^I are deposited in the southern area, and vice versa. Uneven dispersion occurs when large-scale northerly or southerly near-surface winds dominate. These features can be seen in the results of the simulations performed assuming estimated and constant emissions (the hindcast, HC, and long-term, LT, simulations, respectively; the LT simulations assume a fixed release rate during the simulated period), despite the different amounts of material released in these simulations (see the Methods and Supplementary Table S1). This result demonstrates that the dispersion patterns depend strongly on the weather conditions.

The simulated spatial distributions of ^131^I deposition and the wind fields are shown in Supplementary Fig. S1. Both the direction and the range are strongly influenced by the wind conditions. Moreover, the amount of material deposited in the HC simulations differs from that deposited in the LT simulations, particularly from 27 to 30 March (see Supplementary Fig. S1). These results show that the amounts of material dispersed and deposited depend strongly on the timing and amount of radioactive materials released. This result illustrates one reason why the amounts of material dispersed and deposited are quite difficult to estimate without emission source information.

This study presupposes that the numerical model represents the dispersion behaviour of radioactive materials realistically in evaluating long-term (statistical) features. We conduct numerical simulations using a grid spacing of 5 km and the gridded mesoscale model dataset of the Japan Meteorological Agency (JMA) as the boundary conditions (see Methods). Therefore, large-scale to mesoscale weather systems are represented by the simulations. The meridional variations in dispersion are also represented by the simulations (Fig. 1). Furthermore, we have already shown in a previous study^21^ that such experiments represent the diurnal cycle of dispersion. We define the dispersion directions and ranges considering the migration and fluctuation of the large-scale weather systems (see Supplementary Fig. S2). We also assess the dispersion directions of extremely low concentrations (less than or equal to 5% of the total) of radioactive materials in the atmosphere simultaneously; such information would be useful for evacuation planning. For convenience, we define the dispersion directions simulated by the numerical model of the atmosphere as the realistic dispersion directions.

**Figure 2:**
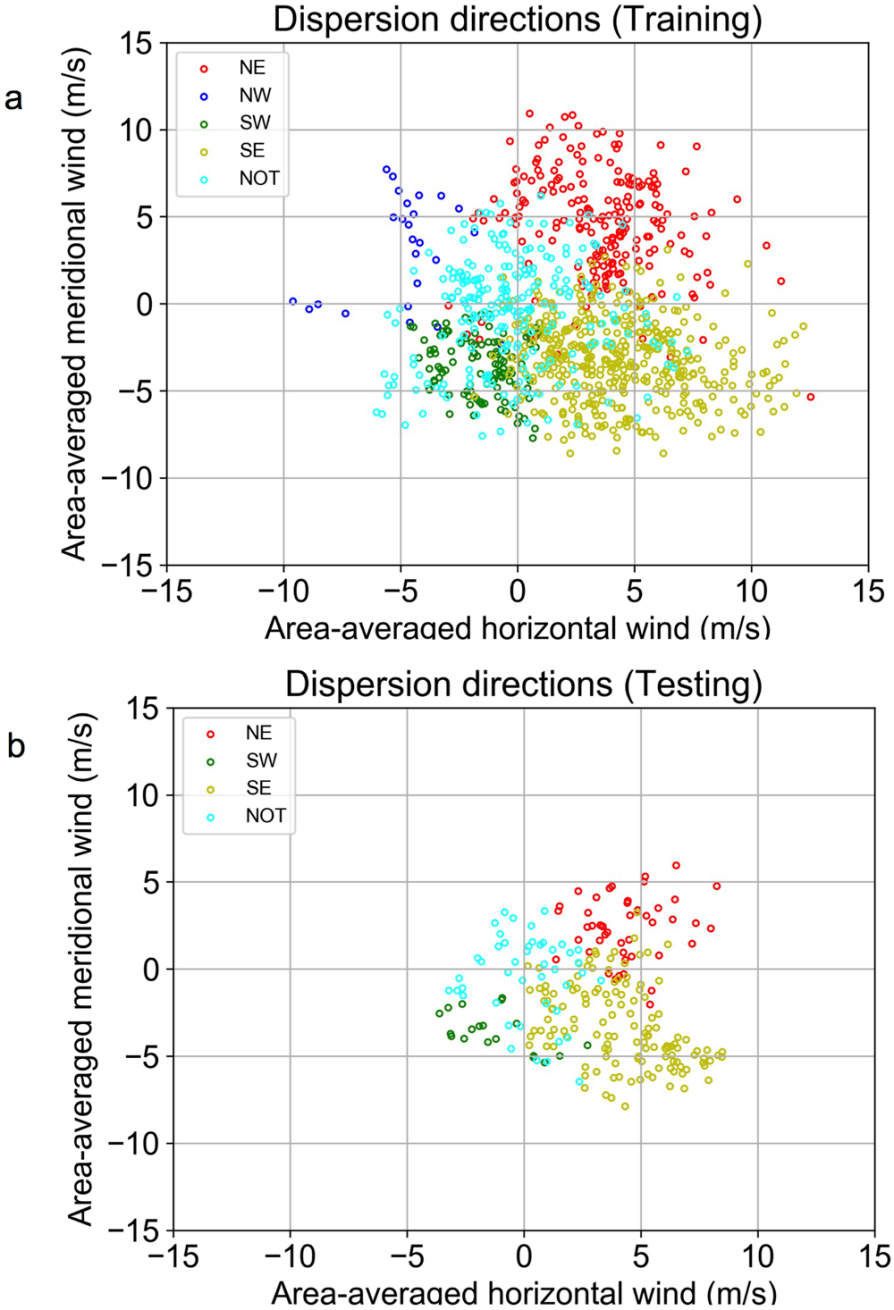
Simulated dispersion directions by area-averaged winds. (**a**) The training data collected in March from 2009 to 2013. (**b**) The testing data collected in March 2011. The colours represent categorized dispersion directions. These graphs were created using Python 3.6.

Here, we assume that the dispersion direction is determined by the patterns of near-surface winds, which affect dispersion directly. A support vector machine (SVM)^22,23^ -based approach is used to verify the hypothesis; this algorithm is trained and tested using previous dispersion directions and the assimilated wind fields (see Methods). We employ two types of assimilated near-surface wind data as feature vectors in the SVM approach. One of these datasets represents the near-surface wind field on a 60 × 60 grid covering the area shown in Supplementary Fig. S2 (FV1). The other dataset is the area-averaged near-surface winds covering the same area (FV2). FV2 estimates the dispersion directions fairly well if the large-scale weather systems are the primary control on dispersion. The relationship between the area-averaged horizontal and meridional near-surface winds (FV2) and the classified dispersion directions can be seen in Fig. 2. The “NOT” means none of them (the other directions) (see Methods).

**Figure 3:**
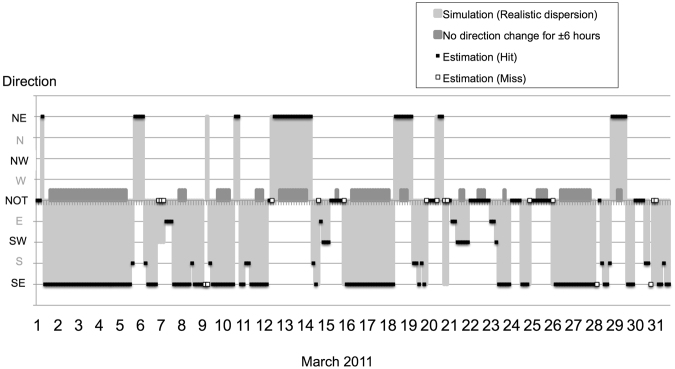
Temporal variations in realistic and estimated directions in March 2011. The realistic dispersion directions are obtained by the dispersion distribution of the output from LT simulation (light shading). The estimated directions are obtained by applying the SVM machine learning method to the 10-m near-surface winds extracted from the MSM-GPV data. The filled square markers and the square markers with white interiors indicate successful and failed predictions, respectively. The dark areas indicate cases in which no change in direction occurred for 6 hours before and after the predictions. This graph was created using Microsoft Excel for Mac 2011.

**Figure 4:**
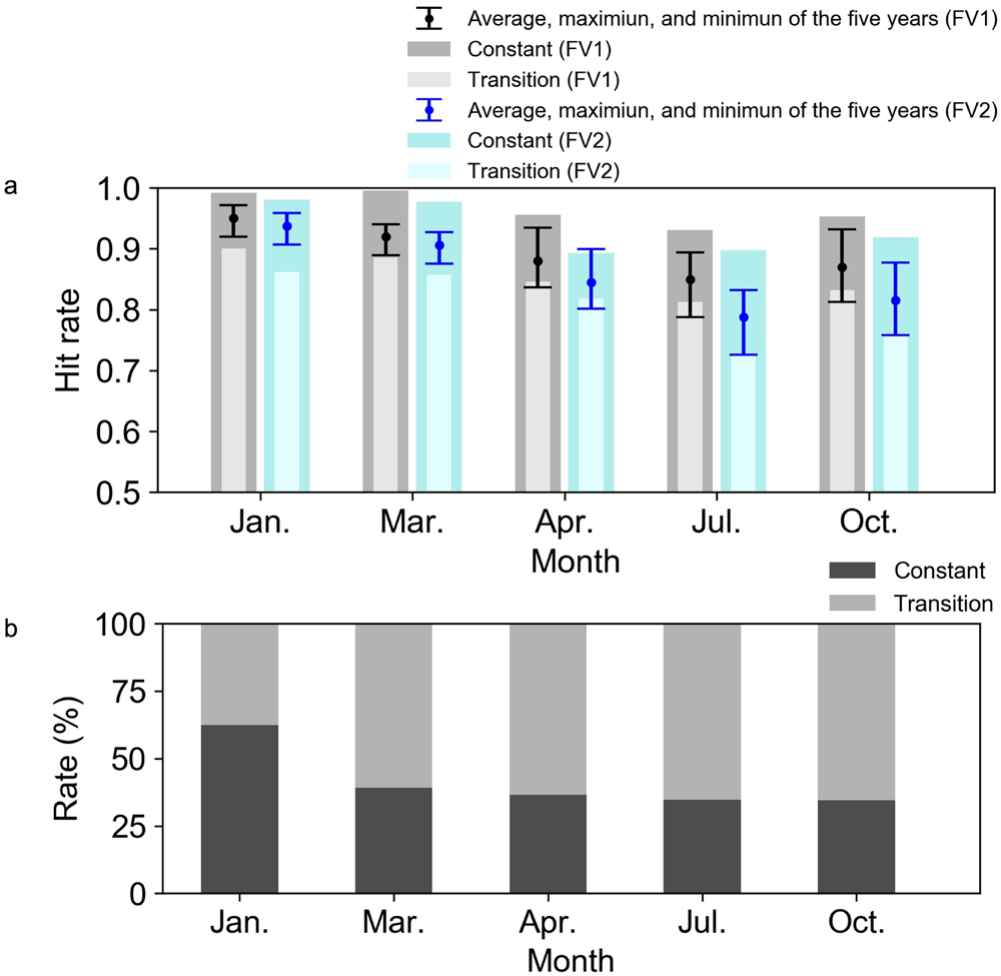
Hit rates and the rates of the Constant and Transition cases. (**a**) Hit rates of FV1 (grey) and FV2 (blue) in January, March, April, July, and October (five-year averaged values). The dark and light shaded bars represent the Constant and Transition states, respectively. The error bars and the black dots indicate the maximum, minimum and average values of the five years from 2009 to 2013. (**b**) The rates of the Constant (dark shading) and Transition (light shading) cases. These graphs were created using Python 3.6.

The temporal variations in the directions estimated by the SVM approach are shown in Fig. 3. The hit rate (see Supplementary Table S2) in FV1 is almost 0.93 in this month. The hit rate in FV2 is almost the same as that in FV1 in January and March (Fig. 4). The miss rates are not found in the Constant cases, in which the dispersion direction does not change from 6 hours before the observation time to 6 hours after. The Constant cases represent those in which large-scale weather systems dominate. The dispersion direction changes substantially in the Transition (non-Constant) cases.

The five-year average hit rates produced by this method exceed 0.85 in the other months and years in FV1 (Fig. 4). High hit rates (exceeding 0.93) are obtained in the Constant cases, whereas the performance decreases somewhat in the Transition cases, particularly in July. The hit rates in FV2 are somewhat lower than those in FV1 in all months. However, the hit rates remain high, exceeding 0.7, in FV2. Moreover, the accuracies in FV1 exceed 0.7 in all months and dispersion directions (see Supplementary Fig. S3).

**Figure 5:**
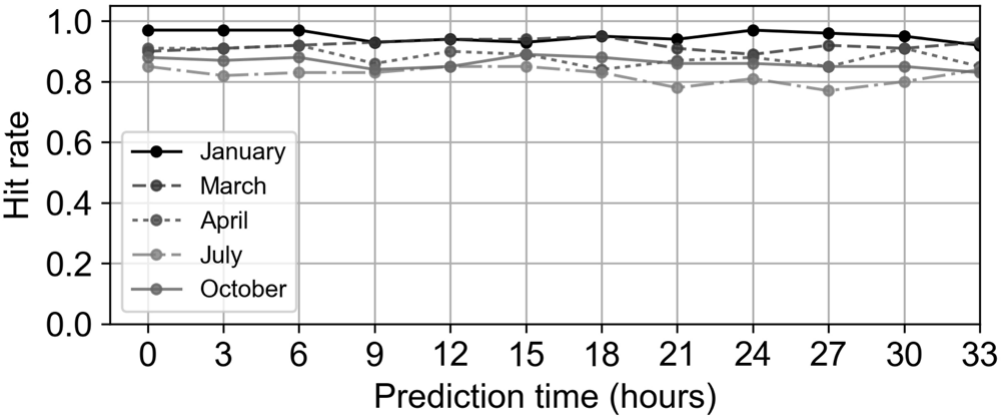
Hit rates of the estimated directions using the forecast winds obtained from JMA. The five-year average hit rates of the estimated directions are shown using the winds forecast from 3 to 33 hours in advance in January, March, April, July, and October. The hit rates at 0 hours indicate the directions estimated using the assimilated winds. The graphs are created using Python 3.

We evaluate the dispersion directions estimated by the SVM approach using the forecast wind fields provided by the JMA to obtain the lead time, which is necessary to prepare for evacuation (see Methods). The hit rates of the estimated dispersion directions for lead times of up to 33 hours exceed 0.77 in all months (Fig. 5). The hit rates increase in winter and exceed 0.92 in January. In the Constant cases, the hit rates exceed 0.95 in January and March, whereas the hit rates are slightly worse (though they still exceed 0.81) in the Transition cases (see Supplementary Fig. S4).

## Discussion

Numerical models cannot reproduce the real world (or the observed distributions of dispersion and deposition) perfectly. Therefore, it is necessary to clarify what the model can reproduce to connect the virtual world (simulation) with the real world (observation). In this study, we regarded the dispersion directions, which are divided into four ranges, as the link to real world. The approximate direction of dispersion can be reproduced because it is based on the principle that the radioactive materials are concentrated downwind of their origins when the prevailing winds blow continuously in one direction. Therefore, the prediction errors could be mostly acceptable if we evaluate the dispersion direction in the wide area.

The dispersion of radioactive materials is greatly influenced by near-surface winds, which are driven by both large-scale weather systems (e.g., the monsoons and extra-tropical cyclones). Furthermore, a previous study reported that nocturnal gravity currents, which are induced by meridional temperature gradients, play an important role in transporting radioactive materials more than 200 km from emission sources^21^. Therefore, the near-surface winds may be the most important factor in the prediction of dispersion directions.

The hit rates in the Constant cases are higher than those in the Transition cases. Large-scale weather systems are found in the composite wind fields associated with the Constant cases (see Supplementary Fig. S5). The wind fields are thought to be well represented by the many similar situations in which these common weather systems appear. On the other hand, the hit rates are somewhat lower in the Transition cases. The wind fields in the Transition cases are not be as well represented as those associated with the Constant cases. This difference may be one of the reasons for the difficulty of the pattern recognition due to the largely changed wind fields associated with the Transient cases.

The performance of FV2 is slightly smaller than that of FV1 in January and March, whereas the differences between FV1 and FV2 are large in April, July, and October (Fig. 4). This result indicates that the area-averaged winds represent a primary control on the dispersion directions in winter, and the effects of smaller-scale wind patterns on the dispersion directions are relatively large in summer. Local winds, such as those driven by diurnal circulation patterns, are thought to be relatively important in the warm season due to the weakened baroclinic instability and westerlies.

The performance metrics obtained for January and March are higher than those in the other months. Thus, we can obtain sufficient generalization ability in winter by training the wind patterns using four years of past data. On the other hand, it appears to be somewhat difficult to obtain high skill in July, although the performance metrics corresponding to each year do not decrease substantially for the five years, and the minimum hit rate seen in FV1 is 0.78. Consecutive misses (incorrect predictions) occur from 19 to 21 July 2011 (see Supplementary Fig. S6). At that time, the wind field was disrupted by a typhoon passing near the southern coast of Japan. The consecutive misses could be caused by the irregular winds produced by the typhoon. In general, the movement of typhoons is quite complex, due to the nonlinearity of atmospheric dynamics^24^. In assessing the skill of this method, it should be recognized that its performance decreases when the wind patterns are disturbed by approaching tropical cyclones (typhoons).

Moreover, disturbances such as typhoons may affect the performance of the dispersion direction predictions made up to 33 hours in advance using the forecast winds (Fig. 5). Relatively low hit rates are found in July. These low hit rates may be due to the disturbances produced by typhoons, in addition to the difficulty of forecasting typhoons in summer. On the other hand, the predictions are almost perfect in the Constant cases in winter, when the winds are controlled by well-observed weather systems, such as the winter monsoon and low-pressure systems caused by baroclinic instability. Moreover, the irregular wind patterns caused by tropical cyclones rarely occur in winter, which also contributes to the high performance of the method in that season.

The performances in the NOT cases could also reflect the diurnal variation patterns. The diurnal wind patterns can be clearly seen in Supplementary Fig. S7. Mesoscale low-pressure systems develop to the east of the Tokyo metropolitan area at night, whereas onshore winds form during the daytime. These features are the same as those seen in a previous study^21^. The local circulation patterns could be the primary cause of the high hit rates. Therefore, the dispersion directions could be assessed by training using the diurnal patterns, which appear frequently in the NOT cases. Clarifying the mechanism of the diurnal circulation would be important in predicting the patterns of migration of radioactive plumes in different areas.

In general, deposition patterns are also strongly influenced by precipitation. However, the performance of the deposition directions is almost the same as that of the dispersion directions (see the Methods and Supplementary Fig. S8). The radioactive materials tend to flow into the central parts of low-pressure systems or convergence zones, which are closely related to the dispersion direction. Rainfall often occurs around those systems. This pattern of behaviour may cause the high hit rates seen in the deposition cases.

We thus confirm the hypothesis that dispersion directions are estimated by the wind patterns corresponding to large-scale weather systems and diurnal circulation patterns in most cases. If this hypothesis were incorrect, the high hit rates and accuracies noted in this study would not be obtained. We also show the levels of performance associated with different conditions (the Constant and Transition cases) and months and provide explanations of the differences in performance in terms of meteorological mechanisms.

The high performance may appear unsurprising because the defined ranges of the dispersion directions are sufficiently large. Given these wide ranges, the model bias is expected to be small. The ranges could also reduce the effects of the movement of weather systems and the fluctuations of mesoscale disturbances on the dispersion directions. Consequently, the degree of dependence on the initial conditions is also expected to be small. Performance typically decreases as the prediction time increases because the prediction errors caused by the sensitive dependence on initial conditions result in increased spread. However, the hit rates do not decrease for forecasts made up to 33 hours in advance (Fig. 5). This result indicates that the adoption of wide ranges of dispersion directions and the high performance of the forecast wind field produced by the JMA could enhance the reliability of the predicted information. If this statement were untrue, the dispersion directions, which are predicted up to 33 hours in advance using the forecast winds, could not be estimated almost perfectly; as noted above, high hit rates exceeding 0.95 are obtained for the Constant cases in winter. The size of training data influences the performance in the machine learning approach. The high performance could also be attributed to the appropriate size of the training data.

The new findings presented in this study include an innovative approach that employs appropriate ranges of dispersion directions and machine learning methods to predict dispersion directions accurately while reducing the uncertainty of numerical models. This approach could be applied to various areas in which wind patterns that correspond to well-observed weather systems, such as monsoons, extra-tropical disturbances, and diurnal local circulation patterns, are detected. Thus, necessary action can be taken, and risk management can be employed to reduce the risk of exposure by considering changing weather conditions and the regional climate in each season. Our findings also provide more reliable information on dispersion directions; in particular, they display reasonable skill for lead times of up to 33 hours, which would be necessary for evacuation, determining when to take potassium iodide and providing warnings regarding the consumption of water intake and the distribution of farm products.

## Methods

### HC simulations

We conduct hindcast (HC) simulations from 11 to 31 March 2011 using the Isotopic Regional Spectral Model (IsoRSM)^25,26^. The height of the emission source is set to the surface because the emission height is estimated to range from 20 to 120 m (fairly close to the surface) in the reverse-estimation method^27–29^. We also use the time-varying release rates estimated by the reverse-estimation method^29^. The mesoscale model grid point value (MSM-GPV) datasets, which are provided by the Japan Meteorological Agency (JMA), serve as the initial and lateral boundary conditions of the model. The spectral nudging method is applied to the lateral boundary data. The simulation domain is shown in Supplementary Fig. S9. The grid spacing is 5 km in this domain. The number of vertical layers of sigma coordinate system is 28. The sigma layer thicknesses in the lower layer (from the surface to the fifth layer) are 0.01, 0.016, 0.02, 0.024, and 0.029, respectively. The horizontal and meridional ranges of the domain are 800 km and 950 km, respectively. A semi-Lagrangian model is used to calculate the transport of radioactive materials^26^.

The wet deposition (washout process) is calculated as:

1 $${\rm{dC}}/{\rm{dt}}=-\,{\rm{\alpha }}\,{\rm{P}}/{\rm{qC}}$$

where C is the atmospheric concentration of radioactive materials; α is the washout coefficient (0.5); and P and q are the water condensation and the water vapour at each atmospheric layer, respectively^30^.

The dry deposition is calculated as:

2 $${{\rm{F}}}_{{\rm{dry}}}={{\rm{V}}}_{{\rm{d}}}{{\rm{C}}}_{({\rm{z}}=1)}$$

where V_d_ is the deposition speed, and C_(z=1)_ is the concentration in the lowest layer. The V_d_ values of ^137^Cs and ^131^I are 1 × 10^−3^ ms^−1^ and 5 × 10^−3^ ms^−1^ over the ocean and 5 × 10^−3^ ms^−1^ and 2.5 × 10^−2^ ms^−1^ over land areas, respectively^31^.

### LT simulations

We conduct long-term (LT) simulations with a fixed release rate in January, March, April, July, and October from 2009 to 2013 (see Supplementary Table S1). The other calculation conditions are the same as in the HC simulation.

### Support vector machine (SVM)

SVM is a supervised learning method and has the advantage that the use of this method to classify patterns for unlearned data maximizes the margin. SVM^32^ -based approaches can be applied to non-linear classification using the Gaussian radial basis function.

3 $$K(x,x\text{'})=\exp (-\sigma \Vert x-x\text{'}\Vert )$$

where *x* and *x*′ are the input vectors of the SVM, and *σ* is the kernel function parameter. The appropriate value of *σ* is automatically estimated by the SVM software^32^. This method, which is called the “kernel trick”, can simplify the calculations associated with this method by replacing the inner products mapped into the high-dimensional feature spaces with a kernel function. We used the “bound-constraint SVM classification” in this study. The SVM approach also supports multi-class classification using the one-against-one method. We use the near-surface winds as the feature vectors and evaluate the performance of the SVM algorithm in this study.

### Classification of the directions of the dispersion of radioactive materials

The SVM algorithm has been used to solve pattern recognition problems^22,23^. We train the SVM algorithm using the simulated dispersion directions in the LT simulation and the gridded assimilated 10-m wind fields obtained from MSM-GPV in the target months from 2009 to 2013, except for the tested year. We first prepare pairs of the categorized dispersion directions (atmospheric concentrations) and the wind fields every three hours in each target month from 2009 to 2013 and produce the SVM-based classifier using some of the pairs (e.g., from 2009 to 2013, except for 2011). We next examine whether the dispersion direction estimated using the wind field and the classifier matches the simulated dispersion direction corresponding to the same wind field, which is taken from the other pairs in the dataset (e.g., March 2011). Finally, the average, minimum, and maximum hit rates and accuracies are calculated in each month using the equations shown in Supplementary Table S2, respectively. The evaluation area is also shown in Supplementary Fig. S2. The assimilated near-surface wind field at 10 m, which has 60 × 60 grid points in the zonal and meridional directions, respectively, is used within the area enclosed within the dashed line (Region A). Radioactive materials do not always spread linearly over a narrow range. In some cases, air masses that contain high concentrations of radioactive materials, which extend widely due to disturbances, are transported by the prevailing winds that accompany large-scale weather systems (see Supplementary Fig. S1). By analysing the atmospheric behaviour of dispersion, we define ranges of potential dispersion directions as primary, secondary, and relatively unaffected so that the method can address the various dispersion patterns and the fluctuations of dispersion due to mesoscale disturbances in the prevailing wind field or the migration of large-scale weather systems (see Supplementary Fig. S2). The atmospheric concentrations in the transported area increase stochastically when horizontally homogeneous prevailing winds dominate, whereas those in the relatively unaffected area decrease, due to ventilation. For example, the dispersion directions are assigned wide ranges because the prevailing winds shift from westerly to northerly, which corresponds to the migration and fluctuations of large-scale weather systems (see Supplementary Fig. S2a–c,e). The wide ranges of those areas could reduce the uncertainty of the dispersion directions simulated by models. We define the dispersion directions by dividing them into four quadrants, specifically “NE”, “NW”, “SW”, and “SE”, using the proportion of the vertically integrated amounts of radioactive materials in each area in the calculation domain of the LT simulations. For example, the dispersion direction is “SE” when the concentration of radioactive materials in the atmosphere in that sector is equal to or greater than 50% of the total and equal to or less than 5% in the opposite direction (NW). The other quadrants, “NE” and “SW”, are defined as the secondary transport areas when the concentrations are less than 50% of the total amounts. The upstream side with a concentration of less than 5% is regarded as the area that is relatively unaffected by the prevailing wind (see Supplementary Fig. S2). In Supplementary Fig. S8, the percentages of material deposited correspond to those of the concentration of radioactive materials in the atmosphere. We decided to use values of 50% and 5% to represent the complex features of the observed and simulated dispersions. This method will also likely be useful for evacuation planning by restricting the dispersion to the upstream side. We also define “NOT” (none of them), which indicates the cases that do not correspond to “NE”, “NW”, “SW”, or “SE”. The relatively unaffected area still occupies the 25% of the entire area, whereas the dispersion range of the primary and secondary areas is 75%. The characterisation of the relatively unaffected area is important for planning evacuations to prevent exposure to radioactive materials. Classifying the wind patterns when the radioactive materials spread along the boundaries is very challenging (see Supplementary Fig. S10a). The radioactive materials often concentrate around the boundary between the “NE” and “SE” regions when cold fronts move to north to south. For that reason, we apply the SVM algorithm to four newly defined regions, “N”, “W”, “S”, and “E”, after applying it to the initially defined sectors (see Supplementary Figs S10 and S11).

### Emission height

The large-scale weather systems form the prevailing wind in the lower troposphere. The wind velocity and direction are slightly changed by the influences of surface conditions, while the wind closes to the geostrophic wind in the upper of the atmospheric boundary layer. The wind velocity would affect the arrival time of the materials at each point. Meanwhile, it was reported that the radioactive materials moved corresponding to the prevailing wind in the lower troposphere in the case of accident at Chernobyl nuclear power plant, even though the maximum of emission height was more than 2,000 m from the surface^2^. Therefore, the dispersion directions would not be largely changed by the emission height in the lower troposphere and the prediction errors could be mostly acceptable in the defined dispersion direction.

### Lead time for evacuation

Preparation time for evacuation is needed when this method is applied to actual accidents or risk management. Therefore, it is necessary to determine how far in advance the method can accurately estimate the dispersion directions using the predicted wind fields, which corresponds to the lead time for evacuation. Here, we investigate the hit rates of the dispersion directions estimated by the SVM approach using the 3- to 33-hour forecast wind fields from MSM-GPV, which is started at 3 UTC every day in this study. For example, the dispersion direction estimated based on the forecast wind field 12 hours after a given time (e.g., 3 UTC on 11 March 2011) is verified using the simulated dispersion direction at the corresponding time (15 UTC, 11 March 2011). The performance of the MSM-GPV forecast is also reflected in the hit rates of this method. We employ the cases with high hit rates as representing the lead time for evacuation that are accompanied by high reliability of the dispersion directions estimated using this method.

### Data availability

The authors declare that all data generated during or analysed during the current study are available from the corresponding author on reasonable request.

### Sample size

No statistical methods were used to predetermine the sample size.

### Code availability

Any codes used in the analysis in this paper and the production of figures can be made available upon request. Please contact T.Y. (takao-y@iis.u-tokyo.ac.jp).

## Electronic supplementary material

Supplementary Information
