# Supplementary material for: Dispersion characteristics of radioactive materials estimated by wind patterns
Source: Sci Rep. 2018 Jul 2;8:9926. doi: 10.1038/s41598-018-27955-4 (PMC6028445; doi:10.1038/s41598-018-27955-4)
Supplement: Supplementary file 1 — Supplementary Information [file 41598_2018_27955_MOESM1_ESM.pdf]

Supplementary information

Dispersion characteristics of radioactive materials estimated by wind patterns

Takao Yoshikane[1], Kei Yoshimura[1]

[1]Institute of Industrial Science, The University of Tokyo, 5-1-5, Kashiwanoha, Kashiwa-shi, Chiba, 277-8574, Japan

Correspondence to T. Yoshikane (takao-y@iis.u-tokyo.ac.jp)

Run type	Nudging	Simulation period	Boundary conditions	Radioactive materials released
HC	ON	11 ~ 31 Mar. 2011	MSM-GPV (6-hourly data)	Estimated values by Kobayashi et al. (2013)
LT	ON	Jan., Mar., Apr., Jul., and Oct. from 2009 to 2013	MSM-GPV (6-hourly data)	A constant amount equal to the maximum value estimated by Terada et al. (2012) during the time-integration constantly

Supplementary Table S1: **Detailed simulation design.** This table was created using Microsoft PowerPoint for Mac 2011.

Contingency Table

Prediction		Observation: YES	Observation: NO
	YES	a	b
	NO	c	d

Hit rate : a/(a+c)
Accuracy (percent correct) : (a+d)/(a+b+c+d)

Supplementary Table S2: **Contingency Table. "**Observation" represents the dispersion directions simulated in the LT simulations, and "Prediction" indicates the dispersion directions predicted by the SVM, which is trained using the simulated dispersion directions and the observed near-surface winds for the preceding four years. This table was created using Microsoft PowerPoint for Mac 2011.

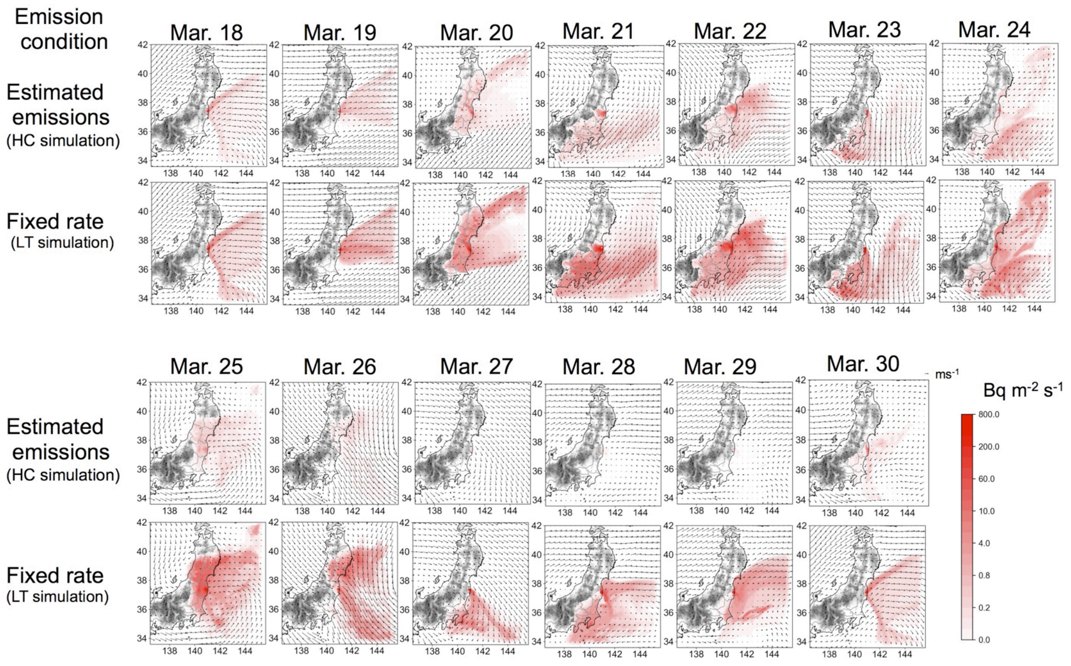

Supplementary Figure S1: **Daily variations in the distribution of radioactive material deposition.** The red shaded areas show the daily deposition of ^{131}I in the HC and LT simulations from 18 to 30 in March 2011. The maps were created using Python 3.6.

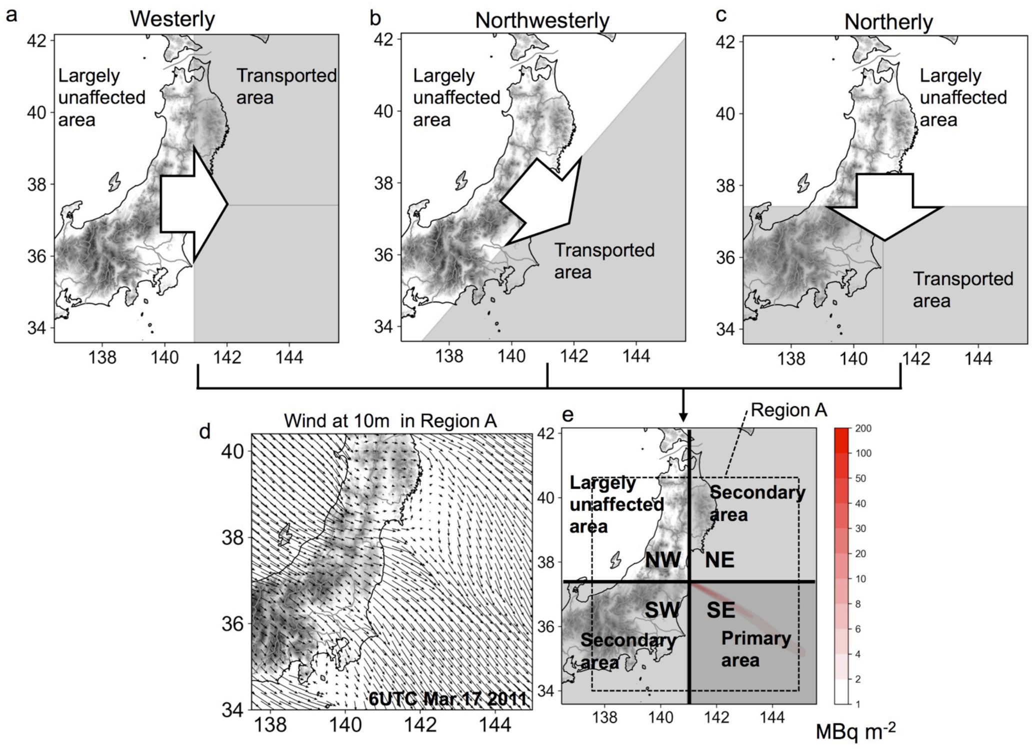

Supplementary Figure S2: **Definition of the dispersion directions using the fundamental characteristics of dispersion patterns.** Simple conceptualization of the areas that are affected and relatively unaffected by radioactive materials due to large-scale, horizontally homogeneous westerly (a), northwesterly (b), and northerly (c) winds. The near-surface winds at 10 m are used for the training and testing of SVM (d). These maps were created using Python 3.6.

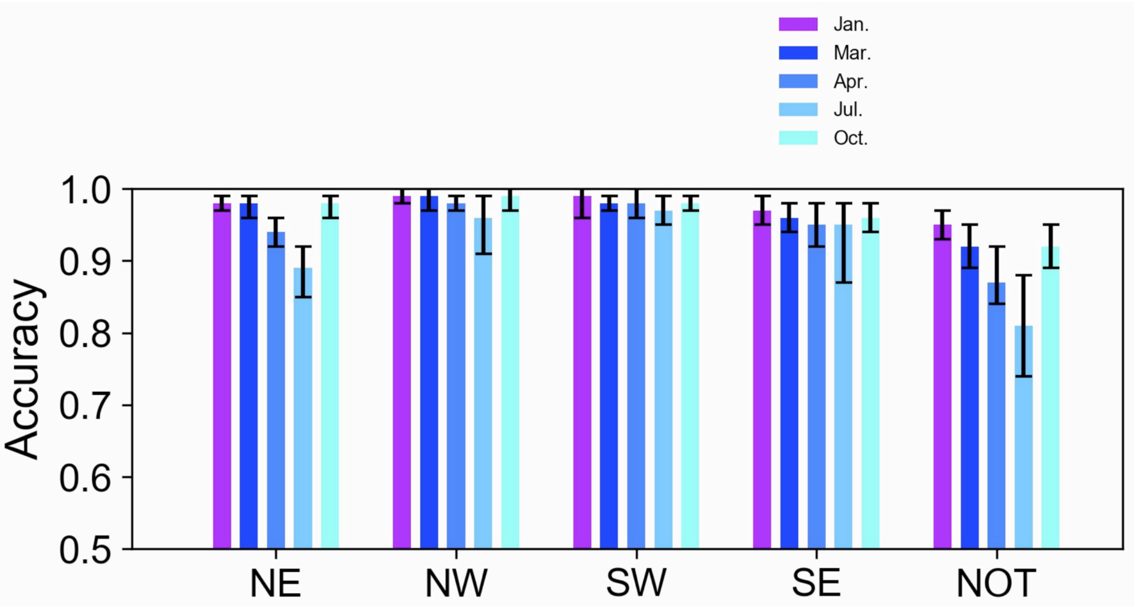

Supplementary Figure S3: **Accuracies of the estimated dispersion directions.** The accuracies were the five-year averaged values and estimated by removing the cases of miss in Division scheme A and hit in Division scheme B conveniently. The error bars are the maximum and minimum values for the five years from 2009 to 2013. The graph was created using Python 3.6.

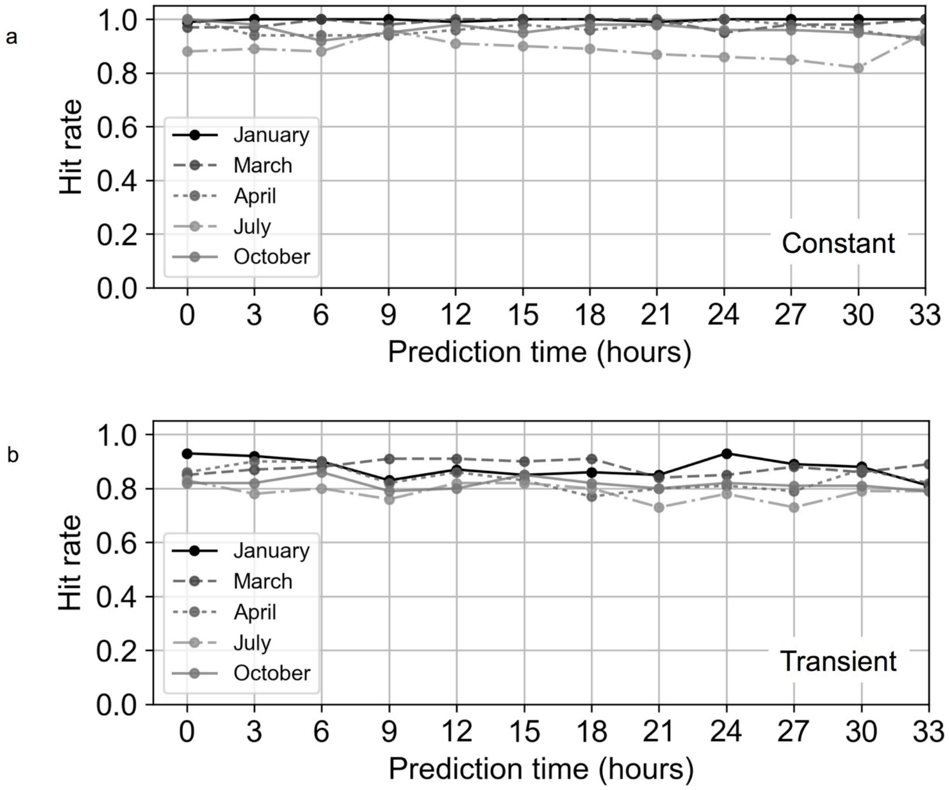

Supplementary Figure S4: **Hit rates of the predicted dispersion directions.** The hit rates are the five-year averaged values. The results for the Constant (a) and the Transient (b) cases are obtained using the winds forecast by JMA. These graphs were created using Python 3.6.

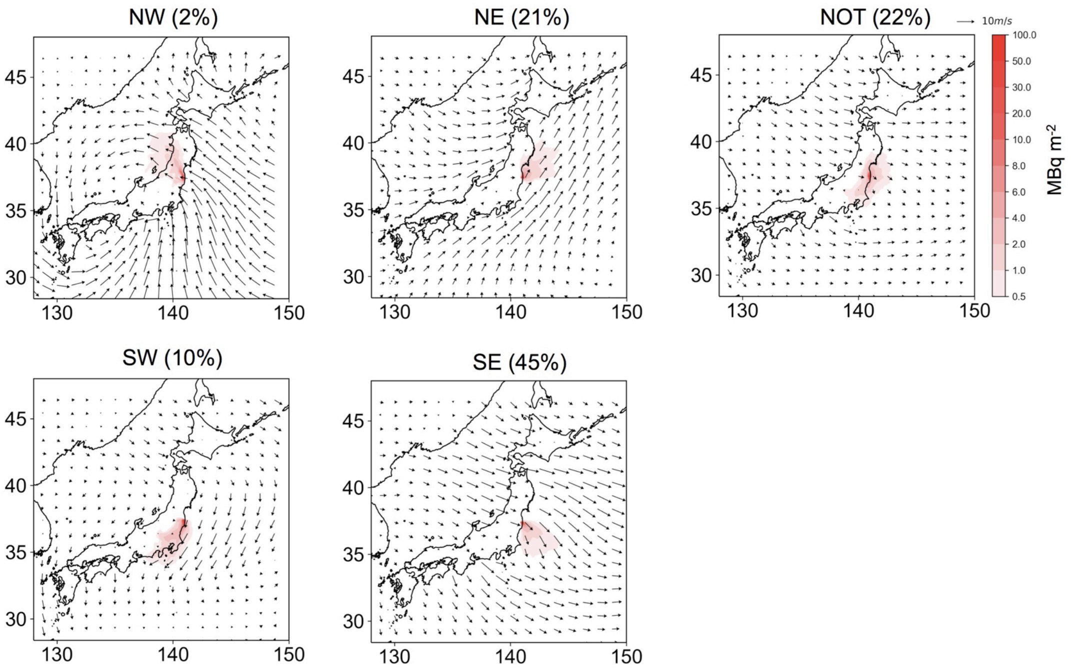

Supplementary Figure S5: **Deposition distributions of ^{131}I and the composite near-surface winds associated with the Constant cases in March.** The percentages indicate the proportions of the different cases for the five years from 2009 to 2013 in the Constant cases in March. These graphs were created using Python 3.6.

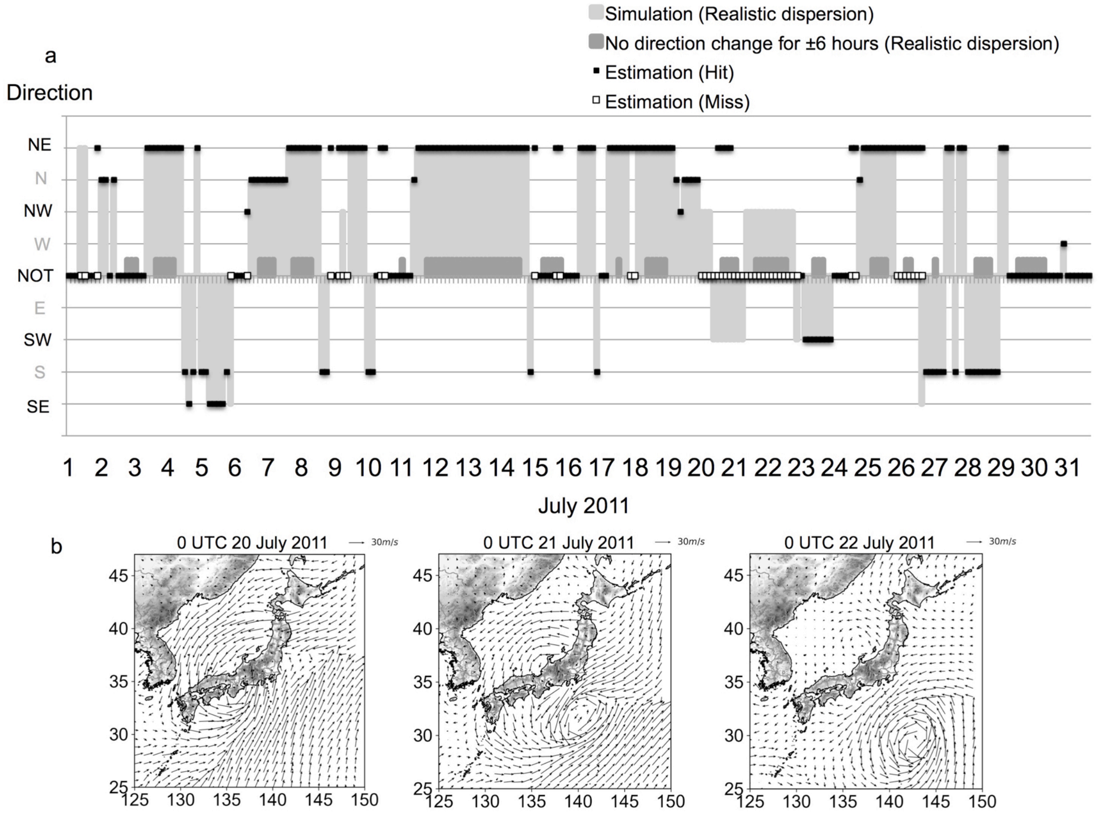

Supplementary Figure S6: **Low hit rate in July 2011 and near-surface winds when a typhoon is approaching.** This figure shows the temporal variations in the simulated and estimated dispersion directions in July 2011 and the near-surface winds from 20 to 22 July 2011. The graph and the maps were created using Microsoft Excel for Mac 2011 and Python 3.6, respectively.

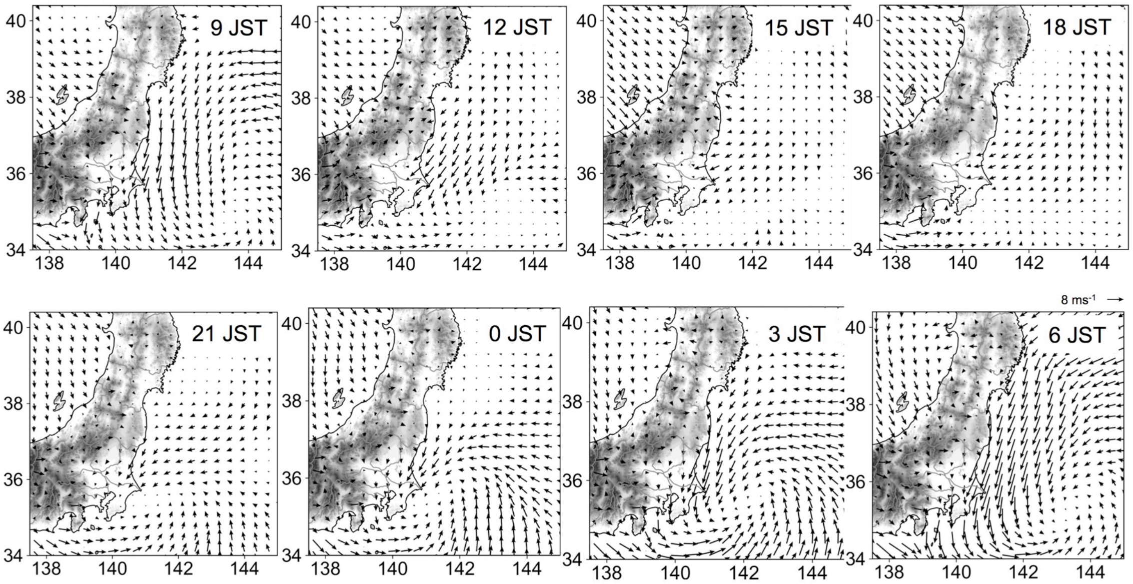

Supplementary Figure S7: **Diurnal changes in the composite near-surface winds in the NOT cases.** A nocturnal mesoscale low forms to the southeast of the Tokyo metropolitan area. These maps were created using Python 3.6.

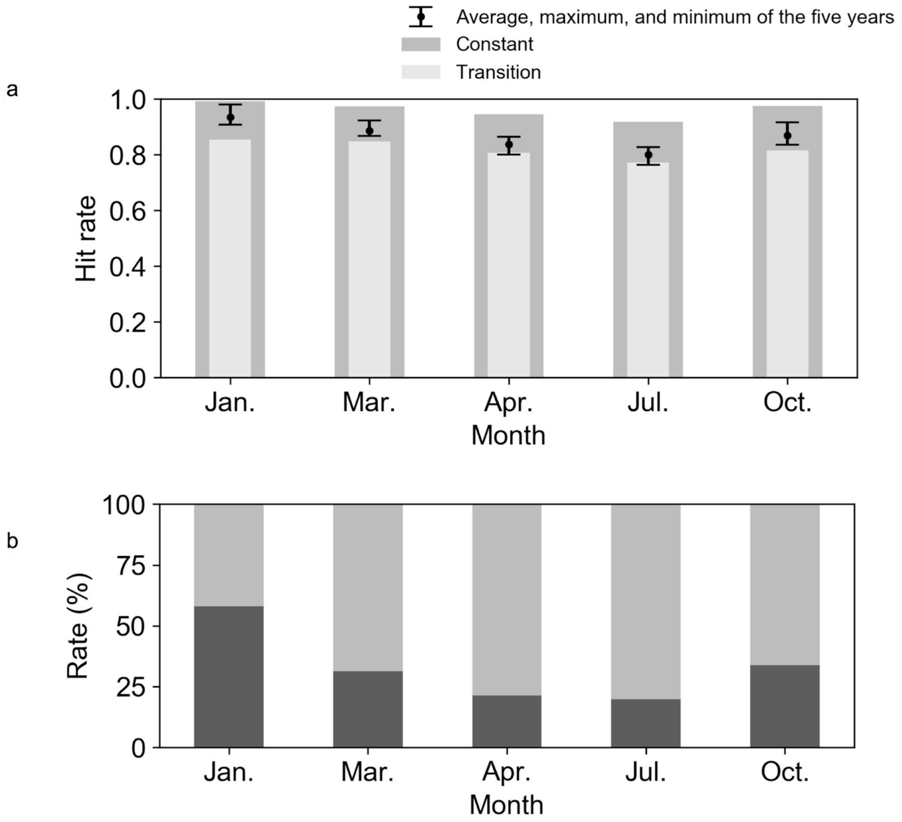

Supplementary Figure S8: **Hit rates and case rates of the total deposition directions of ^{131}I.** The dark and light shaded bars represent the Constant and Transition cases, respectively. The error bars and the black dots are the maximum, minimum and average values for the five years from 2009 to 2013 of each month. The lower panel shows the rates of the Constant and Transition cases in January, March, April, July, and October for the five studied years (a). The rates of the Constant (dark shading) and Transition (light shading) cases (b). These graphs were created using Python 3.6.

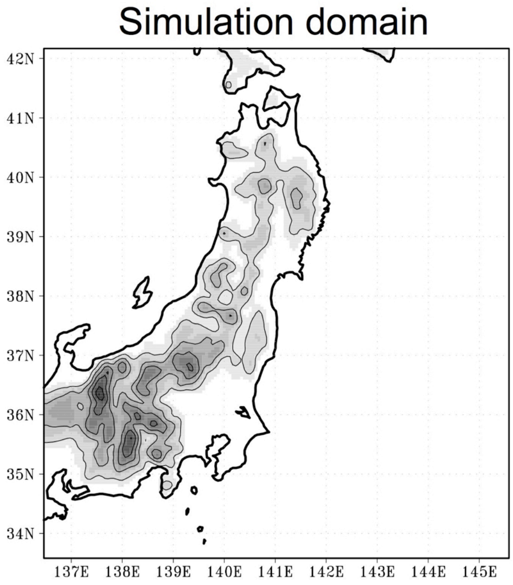

Supplementary Figure S9: **Domain evaluated in the HC and LT simulations.** This map was created using GrADS 2.0.1 (http://cola.gmu.edu/grads/).

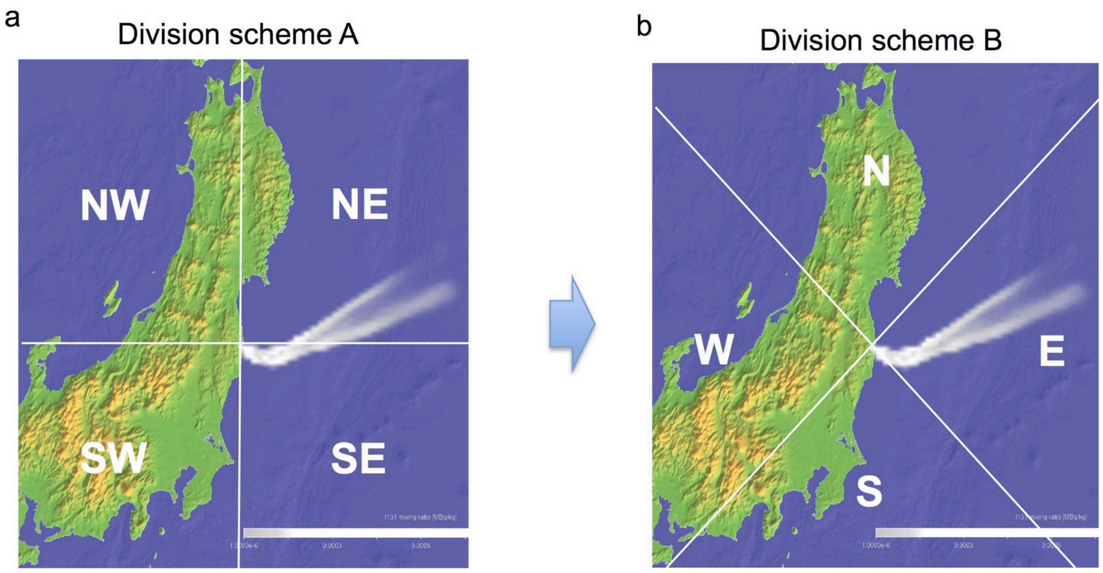

Supplementary Figure S10: **Another definition of the dispersion directions.** Division scheme B (b) is applied when the dispersion directions are missed using division scheme A (a). These maps were created using the Volume Data Visualizer for Google Earth (VDVGE), version 1.1.8 (ESC JAMSTEC; http://www.jamstec.go.jp/ceist/avcrg/vdvge.en.html).

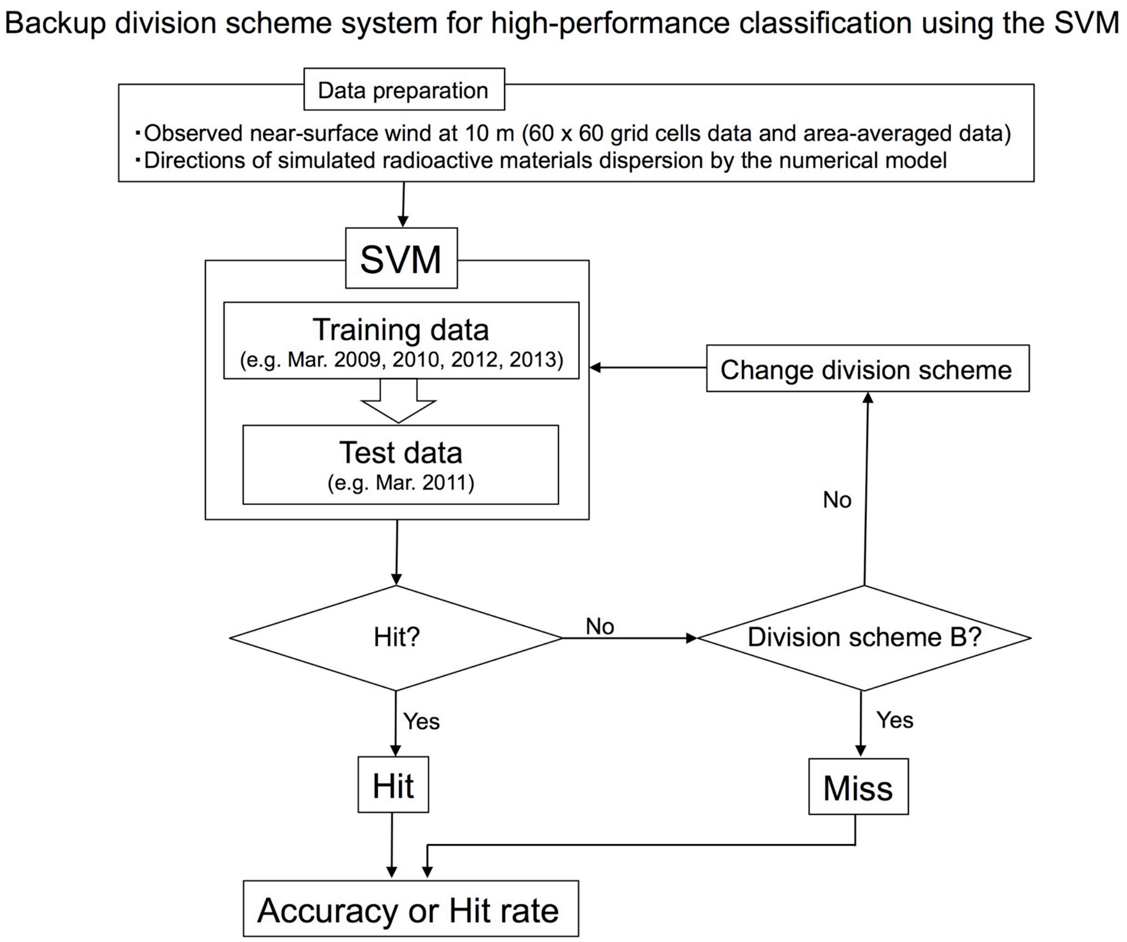

Supplementary Figure S11: **Flow chart of the prediction of dispersion directions using the SVM machine learning method with a backup division scheme.** This flowchart was created using Microsoft PowerPoint for Mac 2011.
